# Triokinase/FMN Cyclase: A Bifunctional Enzyme Linking Metabolism, Stress Responses and Disease

**DOI:** 10.3390/biology15191764

**Published:** 2026-10-03

**Authors:** João Meireles Ribeiro, Fernando Martín-Ruiz, José Carlos Cameselle

**Affiliations:** Departamento de Bioquímica y Biología Molecular y Genética, Facultad de Medicina y Ciencias de la Salud, Universidad de Extremadura, 06006 Badajoz, Spain; fernandomartinruiz14@gmail.com

**Keywords:** triokinase/FMN cyclase, fructose metabolism, triose kinase, flavin metabolism, cyclic FMN, innate immunity, ferroptosis, metabolic stress, inherited disease, alternative splicing

## Abstract

Triokinase/FMN cyclase (TKFC) is a bifunctional enzyme that catalyzes both ATP-dependent triose phosphorylation and the formation of riboflavin 4′,5′-cyclic phosphate (cFMN) from FAD, thereby linking fructose and flavin metabolism through a single protein. Structural modeling supports a model of TKFC as a conformationally dynamic homodimer in which interacting K and L domains cooperate through composite intersubunit active sites to support both kinase and cyclase reactions. Beyond its classical role in fructose metabolism, TKFC has emerged as a determinant of fructose-derived carbon partitioning and has been implicated in nutrient sensing, oxidative stress adaptation, innate immunity, ferroptosis and several inherited human disorders. Recent biochemical and analytical studies have renewed interest in cFMN as a bona fide metabolite and provide evidence consistent with cFMN synthesis and degradation, while its physiological turnover remains to be established. Here, we review the historical development, structural organization, catalytic mechanisms and expanding biological functions of TKFC, highlighting its links with intermediary metabolism, cellular stress adaptation and human disease.

## Simple Summary

Triokinase/flavin mononucleotide cyclase is an unusual human enzyme with two very different activities. This protein was first studied because it helps the body use fructose. We now know that its role is much broader. This review explains how it works, how its two activities are connected, and where it acts in the body. We also examine evidence linking the protein to responses to nutrients and cellular stress, antiviral defense, regulated cell death and inherited disorders. A particularly intriguing feature is its ability to produce a small cyclic molecule involved in flavin metabolism. The biological role of this product remains largely unknown. Taken together, current findings indicate that this protein is more than a simple enzyme of fructose metabolism. It may act as a point of connection between metabolism and other cellular processes. Understanding these links could help explain the molecular basis of several human diseases and provide new insights into how nutrition, metabolism, stress responses and health influence one another.

## 1. Introduction

Triokinase/FMN cyclase (TKFC) is a bifunctional mammalian enzyme that catalyzes both ATP-dependent triose phosphorylation and the conversion of FAD into riboflavin 4′,5′-cyclic phosphate (cFMN) and AMP [1,2]. Initially characterized in the context of fructose metabolism, TKFC occupies a central position in the utilization of fructose-derived trioses through phosphorylation of D-glyceraldehyde and dihydroxyacetone (DHA) [3,4,5]. The subsequent identification of the same protein as the mammalian FAD-AMP lyase (cyclizing) or FMN cyclase (EC 4.6.1.15) unexpectedly linked fructose metabolism and cyclic flavin biochemistry within a single catalytic system [2].

Structural studies of bacterial DHA kinases established a K/L modular organization shared by the ATP-dependent and phosphotransferase system (PTS)-dependent families [6,7,8,9,10]. The crystal structure of the ATP-dependent enzyme from *Citrobacter freundii* revealed an intertwined homodimeric architecture [7]. Later homology modeling and molecular dynamics analyses of human TKFC indicated that this organization is conserved and provided a model for the conformational dynamics underlying catalysis [11,12]. In both the bacterial and the human enzymes, interacting K and L domains cooperate through composite intersubunit active sites [7,11], which in TKFC serve both the kinase and the cyclase reactions [11,12]. The coexistence of two mechanistically distinct activities within the same structural framework has made TKFC an interesting model for understanding bifunctionality, catalytic promiscuity and the evolution of multifunctional metabolic enzymes [13]. Although FAD is the preferred substrate for the cyclase reaction in vitro, TKFC can also catalyze cyclization of other nucleoside diphosphate derivatives capable of analogous intramolecular phosphodiester formation, highlighting a broader catalytic specificity [14].

More recently, the biological relevance of TKFC has expanded considerably beyond classical fructolysis. The enzyme has been implicated in innate immune regulation through modulation of melanoma differentiation-associated protein 5 (MDA5)-dependent antiviral signaling [15,16,17,18,19,20,21], whereas metabolic studies have linked TKFC-dependent pathways to oxidative stress adaptation, nutrient sensing and ferroptosis-associated processes [22,23,24,25,26,27,28]. In parallel, TKFC variants have been associated with multisystem developmental disease, immunological abnormalities, hereditary hypotrichosis and oculocutaneous albinism type 1 [29,30,31,32]. Growing evidence for enzymatic cFMN synthesis and degradation has broadened the conceptual scope of TKFC biology [1,33,34,35].

In this review, we discuss the historical development, structural organization, catalytic mechanisms and expanding biological functions of TKFC. Central to this account is the convergence of metabolic and regulatory functions within a single bifunctional enzyme and its implications for fructose metabolism, cellular stress adaptation and human disease.

## 2. Historical Background: The Convergence of Fructose, Triose Kinase and Flavin Metabolism

The enzymatic activities currently associated with TKFC emerged historically from studies on hepatic fructose metabolism carried out long before the molecular identification of the protein itself (Figure 1). Beginning in the 1950s, classical investigations by Hers and colleagues established the fructose-1-phosphate pathway as the major route of fructose utilization in mammalian liver [3,4]. In this pathway, fructokinase converts fructose into fructose-1-phosphate, which is subsequently cleaved by aldolase B (ALDOB) into dihydroxyacetone phosphate (DHAP) and D-glyceraldehyde. Efficient incorporation of glyceraldehyde-derived carbon into central intermediary metabolism requires ATP-dependent phosphorylation to glyceraldehyde 3-phosphate, a reaction catalyzed by triose kinase. Early biochemical studies therefore identified triose kinase as a component of hepatic fructolysis and characterized its catalytic activity toward D-glyceraldehyde. At that stage, the enzyme was viewed primarily as an accessory carbohydrate-metabolizing activity associated with fructose utilization, without any apparent relationship to flavin metabolism or cyclic phosphate biochemistry.

A key conceptual advance was introduced by Sillero and colleagues, who emphasized that glyceraldehyde generated during fructolysis occupies a central metabolic branch point, the so-called “glyceraldehyde metabolic crossroads” [5]. Decades later, isotope-tracing studies confirmed this classical prediction [27] (Section 5). Thus, modern metabolic studies have largely validated the organization of fructose metabolism inferred from classical hepatic enzymology.

Structural and functional studies of microbial DHA kinases, carried out mainly in the early 2000s, revealed a wider biological significance for triose-phosphorylating systems. DHA kinases from bacteria and yeasts were found to participate not only in carbon assimilation but also in protection against the accumulation of reactive carbonyl compounds [6,7,8,9,10]. In *Saccharomyces cerevisiae*, dihydroxyacetone kinase (DAK)-dependent pathways contribute to detoxification of DHA, a chemically reactive triose capable of promoting glycation reactions and oxidative stress [36]. Such findings suggested that triose kinases may function not only in intermediary metabolism but also in cellular defense and oxidative-stress responses.

A completely independent line of investigation unexpectedly converged with triose kinase biology through studies on unusual flavin metabolites. In the late 1990s, rat liver extracts were found to contain an enzymatic activity capable of converting flavin adenine dinucleotide (FAD) into riboflavin 4′,5′-cyclic phosphate (cFMN) [1]. Subsequent purification and mechanistic characterization demonstrated that this cyclizing lyase or FMN cyclase activity was highly selective and distinct from nonspecific phosphodiesterase reactions. Substrate-specificity studies revealed that the cyclase activity was not strictly restricted to FAD but extended to additional nucleoside diphosphate-X compounds capable of undergoing analogous intramolecular phosphodiester cyclization [14]. The subsequent detection of endogenous cFMN in mammalian liver supported it as a genuine biological metabolite rather than merely an in vitro biochemical artifact [33].

The historical convergence of these apparently unrelated fields occurred when human and rat FMN cyclase activities were identified as ATP-dependent DHA kinases [2]. This finding established the first direct molecular link between fructose metabolism and cFMN formation and provided the basis for later studies on the structure and function of the enzyme. Timson subsequently reviewed the evidence indicating that mammalian triokinase and DHA kinase activities reside in the same protein [37], and the designation TKFC (triokinase/FMN cyclase) was later introduced to reflect the bifunctional nature of the enzyme [11]. This nomenclature was subsequently adopted by the HUGO Gene Nomenclature Committee as the official gene symbol. Before this change, the gene was commonly referred to as *DAK*, based on its sequence similarity to yeast proteins that were initially annotated as dihydroxyacetone kinases. Although subsequent studies have refined the functional interpretation of these proteins, the corresponding yeast genes are still officially designated *DAK*.

Thus, the modern concept of TKFC emerged from the convergence of three historically independent areas of research (Figure 1): hepatic fructose metabolism, microbial DHA kinase biology and mammalian cyclic flavin metabolism. The multifunctional nature of the enzyme reflects this historical trajectory. Structural studies later placed TKFC within a conserved family of ATP-dependent DHA kinases [7,9,10,11], setting the stage for the mechanistic and functional analyses developed in the following sections.

## 3. Chemistry and Enzymology of TKFC Catalysis

The two catalytic activities of TKFC and their distinct cofactor requirements are summarized in Figure 2. This figure provides an overview of the reactions but is not intended for direct comparison of their turnover numbers, because the triokinase and FMN cyclase values shown derive from recombinant human TKFC and native rat-liver TKFC, respectively. Considering the human data separately (Table 1), steady-state kinetic analysis of TKFC at pH 7.5 shows similar *k*_cat_ values for DHA and D-glyceraldehyde (GA) phosphorylation but a markedly lower *K*_m_ for DHA, resulting in a higher catalytic efficiency toward this substrate. Under the same pH conditions, its Mn^2+^-dependent FMN cyclase activity shows an approximately 6-fold lower *k*_cat_ than triose phosphorylation. However, it should be noted that the two activities display distinct pH profiles, and their relative rates vary with pH [11].

A distinctive feature of the FMN cyclase reaction is its divalent-metal dependence. Unlike the triokinase activity of TKFC, which functions as a conventional MgATP-dependent kinase, FMN cyclase activity requires Mn^2+^ or Co^2+^ and is not supported by Mg^2+^. Regarding native liver TKFC, although it is also known to possess Mg^2+^-dependent DHA kinase activity [2], quantitative steady-state kinetic parameters are available only for the FMN cyclase activity (Table 1). Millimolar concentrations of Mn^2+^ or Co^2+^ are required for maximal FMN cyclase activity of the rat enzyme, and the effects of the two metals are strongly pH-dependent, with activity maxima near pH 8.5 for Mn^2+^ and 7.3 for Co^2+^ [14]. Remarkably, these metals also promote the conversion of FAD into cFMN and AMP in the absence of enzyme [38]. Direct comparison under identical conditions shows that, at pH 7.5 and 6 mM metal, the enzymatic reaction proceeds with *k*_cat_ values of 4.8 and 10.7 s^−1^ in the presence of Mn^2+^ and Co^2+^, respectively, whereas the corresponding metal-promoted non-enzymatic reactions have first-order rate constants of 2.1 × 10^−5^ and 1.3 × 10^−5^ s^−1^ [14]. Thus, rat-liver TKFC accelerates FAD cyclization approximately 2.3 × 10^5^-fold with Mn^2+^ and 8.6 × 10^5^-fold with Co^2+^ under these conditions. The parallel between enzymatic and metal-promoted non-enzymatic cyclization supports a direct contribution of the divalent metal to the chemistry of intramolecular phosphate transfer. The substrate specificity of the purified rat-liver enzyme is not restricted to FAD. Several nucleoside diphosphate–X (NDP-X) compounds undergo analogous cyclizing cleavage, with FAD displaying the highest catalytic efficiency and ADP-glucose, a non-physiological compound in mammals, showing an approximately 2-fold lower *k*_cat_/*K*_m_; the remaining substrates are processed much less efficiently [14]. This relatively broad substrate specificity is consistent with interpreting FMN cyclase activity as catalytic promiscuity. Studies of the homologous ATP-dependent DHA kinase from *Citrobacter* sp. further led to the proposal that this promiscuity is modulated by metal switching, with the same catalytic scaffold supporting kinase and cyclase reactions depending on the divalent metal present [13]. The FMN cyclase reaction has also been reproduced using a synthetic bis(Zn^2+^-dipicolylamine) catalyst that converts FAD into cFMN and AMP under near-physiological conditions, providing a biomimetic model of flavin cyclization [39].

Assessment of the physiological significance of this metal dependence is complicated by the absence of metal-binding affinity constants for mammalian TKFC. In addition, total metal concentrations added to in vitro assays cannot be directly equated with intracellular free-metal concentrations, because metal speciation and interactions with cellular or assay components determine effective availability. Nevertheless, the millimolar Mn^2+^ concentrations required for maximal FMN cyclase activity in vitro contrast with the submicromolar concentrations of free Mn^2+^ measured in isolated rat hepatocytes [40]. For context, cytosolic free Mg^2+^ concentrations of approximately 0.37–0.59 mM have been measured in isolated rat hepatocytes [41,42], whereas no comparable cytosolic free Co^2+^ pool has been established in mammalian cells. Fructose overconsumption reduces hepatic manganese content through carbohydrate-responsive element-binding protein (ChREBP)-dependent induction of the manganese exporter SLC30A10, with measurable effects on other Mn-dependent hepatic enzymes [43]. Together, these observations raise the possibility that physiologically relevant changes in intracellular Mn^2+^ availability, including those induced by sustained fructose exposure, may modulate the balance between the triokinase and FMN cyclase activities of TKFC, although direct evidence for such regulation in vivo is currently lacking.

## 4. Structural Organization and Catalytic Mechanism

Bacterial DHA kinases comprise two structurally distinct families. ATP-dependent enzymes, exemplified by that of *Citrobacter freundii*, are homodimers of two-domain subunits [7]. PTS-dependent enzymes, such as that of *Escherichia coli*, use a protein-bound phosphoryl donor rather than ATP and are organized as separate DhaK and DhaL subunits [6,8]. Both families share the K/L modular organization [9,10]. These structurally distinct modules are conventionally designated the K and L domains by analogy with the DhaK and DhaL subunits of PTS-dependent bacterial DHA kinases [6,8,9,10]. Although the letters have sometimes been interpreted as referring to kinase and lyase domains, this is not the origin of the nomenclature. Rather, they simply derive from the names of the bacterial subunits and should not be taken to imply that one domain is responsible for kinase activity and the other for cyclase activity. TKFC belongs to the ATP-dependent family, in which both domains are fused within a single polypeptide chain, and the functional enzyme exists as a homodimer [11]. Accordingly, structural studies of TKFC have largely been interpreted in the context of the ATP-dependent DHA kinase family [7,9,10]. Comparative structural analyses have further suggested that the N-terminal catalytic domain of DAK/TKFC belongs to an evolutionarily conserved phosphotransferase superfamily shared with bacterial EIIA-man and DegV proteins [44]. Although apo and FAD-soaked crystal structures of human DAK/TKFC were reported in a crystallographic conference communication in 2014 [45], no full structural report or Protein Data Bank (PDB) deposition has subsequently appeared.

Figure 3A shows a structural model of homodimeric human TKFC in a near-closed conformation, based on homology modeling [11] using the *Citrobacter* DHA kinase structure [7] as a template, followed by molecular dynamics simulations [11,12] and refinement of a mobile loop [30]. In this model, the two subunits associate in an intertwined arrangement that can be described as L2–K1–K2–L1, with the K domains forming the central region of the dimer and the L domains positioned more laterally. This architecture, first visualized in the *Citrobacter* enzyme, generates two composite active sites per homodimer, formed across subunits: one between K1 and L2 and the other between K2 and L1. Thus, each catalytic center emerges through spatial cooperation between domains contributed by opposite subunits rather than from an isolated monomeric active site. In the triose kinase reaction, the substrates occupy separate regions of the composite active site. The triose substrate, either DHA or D-glyceraldehyde, binds at the K domain, where it forms a covalent adduct with His221, the human residue equivalent to His220 in *C. freundii* DHA kinase [7,11]. ATP binds noncovalently at the L domain [7,9,10,11]. Initial homology models of TKFC placed ATP and DHA too far apart for direct phosphoryl transfer, indicating that productive phosphoryl transfer requires domain closure to bring the L-domain-bound ATP into catalytically competent alignment with the K-domain-bound triose (Figure 3B). Molecular dynamics simulations supported such conformational closure and generated, in one of the two active sites, transient conformations compatible with productive phosphoryl transfer [11,12] (Figure 3C). These findings indicate that catalysis depends on substantial K/L domain movements rather than on a rigid preorganized active site. The simulations further suggest that conformational closure may differ between the two catalytic centers, potentially generating dynamic asymmetry within the homodimer, as described for other conformationally flexible dimeric enzymes [41].

The recent crystal structure of the *Komagataella phaffii* dihydroxyacetone kinase (PpDAK; PDB 26VK) [46] provides the first experimental structural view of a fungal ATP-dependent DAK and an independent structural reference for comparison with human TKFC models. PpDAK is predominantly dimeric in solution and displays the intertwined homodimeric K/L architecture characteristic of ATP-dependent DAKs (Figure 3D). The DHA-binding pocket and catalytic histidine are conserved, although DHA was positioned by docking rather than observed crystallographically. ATP was observed at a surface non-canonical site, whereas its binding at the canonical nucleotide-binding pocket was inferred only by docking. The crystallographically observed ATP at the non-canonical site and the docked DHA are spatially separated and do not define a catalytically productive arrangement. Productive phosphoryl transfer would therefore require ATP binding at the canonical site and/or substantial structural rearrangement. The structural alignment of the model of human TKFC (Figure 3A) with the experimentally determined PpDAK homodimer (Figure 3D) highlights differences in the relative positions of the L domains: crystallized PpDAK adopts a more extended, nearly linear arrangement, whereas the human TKFC model is comparatively more closed (Figure 3E).

Site-directed mutagenesis and analysis of disease-associated human variants of TKFC (Section 10) strongly support this model of K/L domain cooperation [11,29,30,31]. Replacement of His221 abolishes detectable kinase activity while largely preserving FMN cyclase activity, consistent with a role in covalent triose binding. Besides His221, mutagenesis identified Thr112 and Ser446 as additional residues contributing to efficient kinase catalysis, whereas His59 and Asp114 remain structurally proposed catalytic residues awaiting experimental validation [11]. Mutagenesis of Asp401, Asp403 and Asp556 demonstrated that these conserved L-domain residues are required for both triokinase and FMN cyclase activities, consistent with their participation in metal coordination and nucleotide recognition within the composite active site. The mobile loop encompassing residues 535–554 appears particularly important for formation of the catalytically competent closed conformation required for phosphoryl transfer [11,12]. This region contains Arg543, a conserved residue corresponding to an arginine of the L subunit that is required for kinase activity in bacterial DHA kinases [47,48], which approaches the ATP-binding region during domain closure and is essential for productive phosphoryl transfer. Variants affecting this region markedly reduce kinase activity and are associated with human disease [11,29,30,31] (see Section 10). Structural modeling of the C-terminal Gly542Arg substitution illustrates one such mechanism directly: the introduced arginine, adjacent to the catalytically essential Arg543, is incompatible with ATP occupancy and thereby precludes the catalytically competent ATP-bound closed conformation required for phosphoryl transfer [31]. This represents a pathogenic variant that virtually abolishes triokinase activity by preventing productive active-site closure rather than by disrupting substrate chemistry directly.

Mechanistically, the two TKFC reactions differ in kind: triose phosphorylation is intermolecular—a hydroxyl group of DHA or GA attacking the γ-phosphate of ATP—and therefore depends on domain closure to juxtapose the two substrates, whereas FAD cyclization is intramolecular and requires no second substrate and, correspondingly, no comparable domain closure. In docking models, FAD binds through its ADP-like moiety at the L-domain nucleotide-binding region, similarly to ATP, whereas the isoalloxazine portion interacts with the K domain. Competition experiments directly support substantial overlap between the two catalytic systems: ATP strongly inhibits FMN cyclase activity, FAD inhibits triose kinase activity, and D-glyceraldehyde and DHA partially inhibit FMN cyclase activity whereas glycerol, which is not a substrate of TKFC, does not [11]. Together, these observations indicate that the ATP- and FAD-binding regions substantially overlap and that the two reactions compete for access to partially shared composite active-site environments.

Domain-deletion experiments distinguish the structural requirements of the two reactions [11]. Isolated domains are insufficient for kinase activity, confirming that phosphoryl transfer requires the fully assembled K/L catalytic architecture. In contrast, isolated L-domain constructs retain appreciable FMN cyclase activity, although less efficiently than the intact enzyme, suggesting that the L domain contains much of the catalytic machinery required for FAD cyclization whereas interactions with the K domain optimize substrate positioning and active-site geometry. Conversely, the isolated K domain, despite being catalytically inactive, retains its capacity to inhibit MDA5-mediated antiviral signaling [15], a regulatory property examined in detail in Section 7. These findings further support the conclusion that the determinants of triose phosphorylation and FAD cyclization are only partially shared.

Mutational analyses have identified partial separation-of-function variants that can be used to dissect TKFC biology. His221 substitution and the disease-associated Gly542Arg variant abolish or nearly abolish triokinase activity while largely preserving FMN cyclase activity [11,31]. Similarly, the pathogenic Gly192Arg and Arg228Trp variants retain approximately 10% of wild-type FMN cyclase efficiency despite abolished triokinase activity [30]. In contrast, substitutions of Asp401, Asp403 or Asp556 abolish both catalytic activities [11]. These differential effects indicate that the kinase and cyclase reactions share structural determinants but are not mechanistically identical and provide experimentally useful variants for distinguishing the contributions of the two catalytic activities in future cellular studies.

Overall, the available structural and biochemical evidence supports a model in which TKFC functions as a bifunctional homodimer whose two catalytic activities share a composite active-site architecture spanning both domains and subunits but differ in their dependence on K/L domain cooperation. Triose kinase activity requires coordinated K/L domain movements to align a covalent triose intermediate with L-domain-bound ATP, whereas FMN cyclase activity can be partially supported by the L-domain environment alone but is optimized by the intact homodimeric structure. Although the available biochemical, mutational and modeling evidence consistently supports this mechanistic framework, determination of high-resolution human TKFC structures in multiple catalytic states will be important to define directly the conformational basis of K/L domain closure and the distinct structural requirements of the kinase and cyclase reactions. The relative contribution of each catalytic activity in vivo is therefore likely governed by local substrate availability, conformational dynamics and cellular metabolic state—a conclusion with direct implications for understanding how TKFC functions integrate within the metabolic context discussed in subsequent sections. This mechanistic framework also provides the structural basis for understanding the molecular consequences of disease-associated variants affecting active-site geometry or conformational flexibility.

## 5. TKFC in Fructose Metabolism and Metabolic Regulation

In the classical Hers pathway of fructose metabolism [3,4,49], fructose is converted by fructokinase (ketohexokinase, KHK) into fructose 1-phosphate and subsequently cleaved by ALDOB into DHAP and D-glyceraldehyde. The latter is phosphorylated by TKFC in an ATP-dependent reaction to generate D-glyceraldehyde 3-phosphate, thereby enabling the direct incorporation of fructose-derived carbon into central glycolytic and gluconeogenic pathways (Figure 4). This position places TKFC at a critical branch point in fructose metabolism. Classical biochemical studies by Sillero and colleagues identified fructose-derived glyceraldehyde as the center of the “glyceraldehyde metabolic crossroads”, a metabolic framework in which triose kinase displays the lowest *K*_m_ among the competing enzymes, indicating that phosphorylation is strongly favored under physiological conditions [5]. Isotopic studies by Hue and Hers confirmed the physiological importance of this route, demonstrating that triose phosphorylation predominates during hepatic fructose metabolism and provides most of the carbon incorporated into glycogen, whereas oxidative glycerate pathways make only a minor contribution [50]. Consistent with the central metabolic role of glyceraldehyde, classical physiological studies showed that glucose-stimulated insulin secretion was markedly impaired in pancreatic islets from fasted rats, whereas secretion induced by D-glyceraldehyde remained largely preserved, indicating that the fasting-induced metabolic defect occurred upstream of triose metabolism [51].

Modern metabolic and isotope-tracing studies have substantially reinforced this classical framework. Liu et al. demonstrated that loss of TKFC redirects fructose-derived carbon away from lipogenesis and toward pathways associated with cellular stress responses. In hepatocytes lacking triose kinase activity, fructose oxidation increased, oxidative stress and cell death were enhanced, and glyceraldehyde metabolism shifted toward aldehyde dehydrogenase-dependent glycerate formation and serine biosynthesis [27]. Similarly, genetically divergent mouse strains resistant to fructose-induced hepatic steatosis showed lower intestinal *Tkfc* expression, a higher *Aldh1a1/Tkfc* expression ratio, and increased production of fructose-derived glycerate [52]. Collectively, these findings identify TKFC as an important determinant of fructose carbon partitioning, linking the classical concept of the glyceraldehyde metabolic crossroads to contemporary views of metabolic stress adaptation.

Extending this metabolic framework to another tissue, isotope tracing with [U-^13^C]fructose in ex vivo working mouse hearts demonstrated active myocardial fructose metabolism, with substantial labeling of glyceraldehyde and glycerate and preferential glycerate production relative to glucose. Although TKFC expression or activity was not examined, these findings indicate that the glyceraldehyde metabolic crossroads is also operative in the heart and suggest that fructose carbon partitioning toward the oxidative glycerate branch may be tissue-dependent [53].

A further signaling dimension has recently been added to this metabolic crossroads. Glycerol 3-phosphate, which can be generated from fructose-derived DHAP or through the sequential conversion of glyceraldehyde to glycerol and glycerol 3-phosphate, was identified as a high-affinity ligand of the ChREBP glucose-sensing module and induced a transcriptional program that included *Fgf21* and *Tkfc* [54]. These findings raise the possibility of a feedback relationship: TKFC-dependent carbon partitioning could influence the amount of fructose-derived carbon reaching glycerol 3-phosphate, whereas glycerol 3-phosphate-activated ChREBP could in turn increase *Tkfc* expression. *Fgf21* represents another output of the same transcriptional program, but neither this proposed feedback relationship nor a direct role of TKFC in its regulation has been experimentally tested.

The recognition that the small intestine acts as a major site of fructose catabolism has substantially revised current views of dietary fructose metabolism [55,56]. Under physiological fructose loads, much of the ingested fructose is metabolized within enterocytes into glucose, lactate and organic acids before reaching the liver [55]. Recent spatial metabolomic analyses have refined this intestinal-first model by demonstrating that fructose utilization is regionally organized along the crypt–villus axis, with the highest fructose catabolic activity occurring in villus enterocytes [57]. This extends the physiological relevance of fructose-derived triose metabolism and suggests that TKFC-mediated glyceraldehyde phosphorylation may contribute to fructose clearance at multiple tissue sites beyond the liver. Recent evidence extends this view to brown adipose tissue, which takes up circulating fructose and expresses KHK, ALDOB and TKFC at moderate protein levels. Fructose uptake by brown adipose tissue increases more than 2-fold during cold adaptation, indicating that peripheral fructose utilization can be dynamically recruited according to physiological state [58]. Transcriptomic evidence also extends *TKFC* expression to the lactating mammary gland, where *TKFC* was differentially expressed between sheep breeds with contrasting lactation phenotypes and contributed to enrichment of the carbon-metabolism pathway [59]. Spatially resolved transcriptomic and proteomic analyses point to a role for TKFC in the metabolic compartmentalization of the liver. *Tkfc* transcript and TKFC protein show preferential periportal expression in mouse liver and *Tkfc* was identified among genes showing concordant distribution of transcript and protein expression across the liver lobule, consistent with fructose-derived triose metabolism being integrated into the functional organization of this hepatic unit [60,61]. Proteomic analysis of murine hepatocytes spatially separated by fluorescence-activated cell sorting (FACS) further supported this zonation pattern, showing a progressive portal-to-central decline in TKFC abundance [62].

Fructose is not exclusively derived from dietary sources but can also be generated endogenously from glucose through the polyol pathway. In the liver, glucose-induced accumulation of endogenous fructose and its KHK-dependent metabolism have been implicated in the development of metabolic dysfunction [63]. Once formed, endogenous fructose can enter the canonical Hers pathway and may therefore provide an additional source of glyceraldehyde for TKFC-dependent metabolism, although the specific contribution of TKFC to this endogenous flux has not been quantified.

In addition to glyceraldehyde, TKFC phosphorylates DHA to DHAP [2,11], providing a route for the metabolic clearance of this reactive triose. The possibility that this activity also contributes to cellular defense against reactive triose and carbonyl stress, and that DHAP may act as a signaling metabolite linking triose metabolism to nutrient sensing, is discussed in Section 8.

Fructolytic enzymes including TKFC participate in transcriptional responses associated with feeding and metabolic remodeling. Fructose-induced upregulation of intestinal *Khk*, *Aldob* and *Tkfc* mRNAs and KHK, ALDOB and TKFC proteins requires both glucose transporter 5 (GLUT5)-mediated fructose uptake and KHK-dependent metabolism [64]. Increased jejunal *Tkfc* expression at both the mRNA and protein levels has also been observed after prolonged high-fructose feeding in adult mice [65]. ChREBP-dependent feed-forward regulation of fructolytic genes, including *KHK*, *ALDOB*, *TKFC* and *SLC2A5* (GLUT5), has subsequently been proposed to facilitate adaptation to sustained fructose exposure [66]. In line with this model, Tsukamoto et al. showed that hepatic *Tkfc* expression is strongly induced during refeeding and is directly regulated by both ChREBP and hepatocyte nuclear factor 4α (HNF4α). Functional promoter analyses revealed cooperative interactions between ChREBP- and HNF4α-responsive elements, providing mechanistic support for the inclusion of *Tkfc* within this fructose-responsive transcriptional network [67]. More recently, integrated transcriptomic and metabolomic analyses identified *Tkfc* among the fructolytic genes most strongly downregulated following liver-specific suppression of ChREBP, together with *Khk*, *Aldob* and *Pklr* [68]. This positions TKFC within a metabolic program coordinating fructose utilization with hepatic energy homeostasis. Evidence for a wider role in metabolic adaptation is also provided by transcriptional and functional studies linking TKFC to oxidative and inflammatory stress. *Tkfc*, together with *Khk*, *Sord* and *Hnf4a*, was identified among direct nuclear factor erythroid 2-related factor 2 (NRF2)-regulated genes induced during arsenic-associated diabetogenic metabolic remodeling, linking activation of fructose metabolic pathways to increased gluconeogenesis and hepatic glucose output [69]. Subsequent studies implicated TKFC-associated pathways in inflammatory signaling and metabolic disease, including fructose-induced podocyte injury and experimental metabolic dysfunction-associated steatotic liver disease (MASLD) models [70,71]. Together, these results place TKFC within regulatory networks that coordinate fructose metabolism with cellular and physiological adaptations. Consistent with these observations, comparative liver proteomic analysis also demonstrated increased hepatic TKFC abundance after chronic saturated-fat feeding, whereas replacement with polyunsaturated-fat diets, particularly those with a lower ω6/ω3 ratio, reduced TKFC expression [72], further supporting the integration of TKFC into nutrient-responsive metabolic programs. Consistent with pharmacological modulation of fructose metabolism, urinary proteomic profiling also identified reduced TKFC abundance after metformin treatment in rats, although the tissue origin and mechanistic basis of this change remain unknown [73].

The integration of TKFC within these regulatory networks also has pharmacological implications. Park et al. demonstrated that KHK depletion and pharmacological inhibition of KHK catalytic activity produced markedly distinct metabolic and transcriptional effects in vivo, including divergent consequences for lipogenesis, fatty acid oxidation and glycogen accumulation. Importantly, the KHK inhibitor also partially inhibited TKFC activity and reduced hepatic TKFC expression [74], consistent with fructose metabolism operating as an integrated regulatory network rather than a simple linear pathway.

A central methodological challenge in interpreting TKFC function is the difficulty of distinguishing intrinsic catalytic defects from systemic pathway-level metabolic effects, a problem well illustrated by the common Ala185Thr substitution. Metabolic flux analyses in hepatocytes led to the proposal that the Thr185-containing form behaves functionally as a “null” enzyme in the context of fructose-driven lipogenesis [27]. However, direct triokinase activity was not assessed in that study. Subsequent biochemical analyses demonstrated that the Thr185-containing enzyme is fully active as a triokinase both in vitro and in yeast complementation assays and is catalytically indistinguishable from the Ala185 form [75]. Interpretation of this substitution is further complicated by its population frequency: according to gnomAD [76], the Thr185 allele occurs at a global frequency exceeding 0.9, indicating that the Thr185-containing protein represents the predominant human form rather than a rare variant. The apparent discrepancy between metabolic phenotypes and direct enzymatic measurements therefore most likely does not reflect an intrinsic catalytic deficiency. Regulatory or cellular-context effects may explain this discrepancy, but this remains to be tested directly. More broadly, the Ala185Thr case also provides an important methodological caution for the interpretation of variants in multifunctional proteins. A cellular or metabolic phenotype should not be assumed to reflect loss of a specific catalytic activity unless that activity is directly measured, particularly when the protein has multiple catalytic and non-catalytic functions. The high population frequency of Thr185 further argues against interpreting this common variant as intrinsically deleterious without direct functional evidence.

Recent evidence suggests that hepatic fructose metabolism is also subject to extracellular regulation. Zhao et al. demonstrated that hepatocyte-derived angiopoietin-like protein 3 (ANGPTL3) promotes hepatic fructose metabolism through phosphoinositide 3-kinase/protein kinase B (PI3K/AKT) signaling while coordinately regulating the expression of the hepatic fructose transporter GLUT8 and key fructolytic enzymes, including KHK and ALDOB [77]. Although TKFC was not examined, these findings reveal an additional extracellular regulatory layer controlling hepatic fructose uptake and fructolytic capacity. Such integrated regulation may contribute to context-dependent control of fructose flux through the Hers pathway.

## 6. Cyclic FMN: Formation, Enzymatic Metabolism and Evidence for Physiological Turnover

Canonical mammalian flavin cofactor biosynthesis proceeds through two sequential enzymatic reactions: the phosphorylation of riboflavin to form FMN, catalyzed by riboflavin kinase, followed by the adenylation of FMN to generate FAD, catalyzed by FAD synthase [78]. Against this well-established biosynthetic background, cFMN represents an additional, structurally unusual product of FAD metabolism whose physiological significance remains poorly understood [1,14]. As discussed in Section 3, cFMN can be formed by metal-promoted non-enzymatic cyclization of FAD, whereas TKFC greatly accelerates the same conversion to cFMN and AMP in vitro [14,38]. These established in vitro reactions should be distinguished from the separate question of whether TKFC-mediated cFMN formation participates in a physiological turnover pathway in vivo. A schematic overview of the current evidence and proposed relationships is presented in Figure 5.

Further evidence comes from the direct detection of endogenous cFMN in rat liver at nanomolar concentrations [33], the identification of a rat liver cyclic phosphodiesterase activity capable of hydrolyzing cFMN to 5′-FMN [1], the demonstration that purified mammalian phosphodiesterases, including PDE4 and PDE6, can hydrolyze cFMN to 5′-FMN in vitro under physiological aqueous conditions [34], and more recent analytical studies identifying cFMN as a detectable intermediate of FAD metabolism [35]. These findings establish that cFMN occurs in mammalian tissue and that mammalian enzymatic activities capable of both its formation and degradation have been demonstrated, supporting a potential, but not yet demonstrated, cFMN turnover pathway.

Available evidence indicates that cFMN is present in phylogenetically diverse organisms. Chromatographic studies identified riboflavin 4′,5′-cyclic phosphate not only in mammalian tissues but also in baker’s yeast and several food products including fermented dairy products and liver extracts [79,80]. These studies also raised the possibility that cFMN may have been misidentified as FMN because of chromatographic overlap and the limited resolution of older analytical methods. cFMN was also identified as the naturally occurring autofluorescent compound of the brown alga *Scytosiphon lomentaria* [81]. These observations demonstrate that cFMN is not restricted to mammalian tissues but do not establish how it is formed in these biological systems.

The occurrence of cFMN in mammalian tissues, fungi, fermented foods and algae raises intriguing evolutionary and physiological questions regarding the origin and biological significance of flavin cyclization pathways. The coexistence of enzymatic systems mediating both cFMN synthesis and hydrolysis is compatible with a biological role for cFMN, but does not by itself establish that these activities form a regulated metabolic pathway in vivo. Moreover, because FAD can undergo metal-promoted non-enzymatic cyclization [14,38], the detection of cFMN in biological samples does not establish an enzymatic origin. Even if TKFC contributes to cFMN formation in vivo, its FMN cyclase activity may represent catalytic promiscuity of an ancestral kinase scaffold rather than a coevolved physiological function [13]. Addressing these issues will require determining whether TKFC contributes to cFMN formation in vivo and whether cFMN has specific physiological targets or functions. One possibility is that conversion of FAD into cFMN transiently modifies flavin availability, reactivity or intracellular compartmentalization under defined metabolic or stress conditions. In this context, the distinctive structural and photochemical properties of cFMN hint that flavin cyclization may influence signaling or photobiological processes in specialized cellular environments, although no specific cFMN-binding partners or physiological effects have yet been identified.

Resolving these questions will likely require sensitive metabolomic approaches capable of monitoring cFMN dynamics in vivo. Recent methodological advances, including chemically validated cFMN standards and liquid chromatography–mass spectrometry (LC-MS) workflows capable of distinguishing cFMN from FMN, now provide the analytical tools needed for reliable quantification of endogenous cFMN in biological samples [35]. Combined with separation-of-function TKFC variants that differentially affect its two catalytic activities, these approaches should facilitate direct evaluation of the contribution of TKFC to cFMN formation in vivo and help determine whether cFMN participates in a physiological turnover pathway.

## 7. TKFC and Innate Immunity

A major expansion of TKFC biology emerged when the protein was identified as a negative regulator of MDA5-mediated antiviral signaling. MDA5 and retinoic acid-inducible gene I (RIG-I) are the two principal signaling members of the RIG-I-like receptor family, closely related cytosolic sensors of viral double-stranded RNA [16] that share a common domain architecture and converge on mitochondrial antiviral-signaling protein (MAVS)-dependent activation of the IκB kinase ε/TANK-binding kinase 1/interferon regulatory factor 3 (IKKε/TBK1/IRF3) axis to induce type I interferon [17]. Despite this close relatedness, the two receptors discriminate between distinct RNA ligands, with RIG-I recognizing short blunt-ended 5′-triphosphorylated double-stranded RNA and MDA5 recognizing long double-stranded RNA [18]. Unlike RIG-I-dependent pathways, MDA5-mediated interferon activation was selectively suppressed by TKFC, which was reported to interact directly with MDA5 and to inhibit downstream antiviral signaling under basal conditions [15]. This suggested that TKFC may function not only as a metabolic enzyme but also as a modulator of cytoplasmic RNA-sensing pathways. Additional mammalian support came from studies of dengue virus (DENV) antibody-dependent enhancement, in which *DAK* expression was maintained in DENV–antibody-complex-infected monocytic cells while RIG-I/MDA5 signaling and type I interferon production were suppressed; suppression of the RIG-I/MDA5 pathway was also observed in peripheral blood mononuclear cells from patients with severe secondary dengue infection [82]. Although DAK was not functionally manipulated in that study, these findings are consistent with involvement of DAK/TKFC in virus-associated attenuation of cytoplasmic RNA sensing. Other mammalian studies have provided systems-level and disease-associated support [31,83], whereas direct regulation of the TKFC–MDA5 interaction was subsequently demonstrated in a disease-specific context [21] (see below).

Later studies in teleost fish supported evolutionary conservation of this immunomodulatory activity. In miiuy croaker, inducible microRNA-122 modulated antiviral signaling through regulation of *DAK*/*TKFC* expression after polyinosinic:polycytidylic acid [poly(I:C)] stimulation [19], whereas studies in black carp reported that DAK inhibits MDA5-mediated antiviral signaling similarly to mammalian TKFC [20]. Functional validation outside mammalian systems remains limited; nonetheless, these observations are consistent with the relationship between TKFC and innate immune regulation reflecting a conserved biological association rather than an isolated mammalian phenomenon. In line with these observations, a human systems-level analysis identified TKFC within an *IFIH1* (MDA5) functional-association network, although no direct functional role of TKFC was examined [83].

The physiological significance of these observations is reinforced by the central role of the *IFIH1* (MDA5) pathway in antiviral immunity and inflammatory disease. Genetic and immunological studies have demonstrated that both gain- and loss-of-function alterations in *IFIH1* are associated with severe human disorders, including chronic interferonopathies, autoinflammatory syndromes and increased susceptibility to respiratory viral infections [84,85,86]. Experimental overexpression of MDA5 has likewise been shown to induce chronic type I interferon responses and to exacerbate autoimmune phenotypes in susceptible genetic backgrounds [87]. More broadly, the later recognition of *IFIH1* (MDA5) dysregulation as a defining component of type I interferonopathies further underscored the importance of cellular mechanisms that restrain basal antiviral signaling and preserve self/non-self discrimination in nucleic acid sensing pathways [88].

Current evidence points to protein–protein interaction networks as the primary basis of TKFC immunomodulatory activity, rather than direct catalytic mechanisms. Notably, the isolated K domain, despite being catalytically inactive [11], retains inhibitory activity toward MDA5 signaling [15]. This finding indicates that at least part of the immunomodulatory function of TKFC depends on structural determinants distinct from those required for triokinase or FMN cyclase activity and suggests a partial functional separation between catalytic and regulatory properties within the protein. A question is raised by the immunodeficiency-associated Gly542Arg variant, which lies in the C-terminal L domain, whereas MDA5 binding maps to the N-terminal K domain. Based on the modeled intersubunit architecture, a C-terminal lesion could, in principle, propagate conformational effects to the N-terminal MDA5-interaction surface [31]. However, this possibility remains speculative and has not been experimentally demonstrated.

A subsequent study identified a disease-relevant regulatory layer acting on this interaction: circSOBP, a circular RNA downregulated in glioma, was found to bind TKFC and to disrupt its interaction with MDA5, thereby de-repressing MDA5-dependent activation of the IKKε/TBK1/IRF3 axis and type I interferon transcription [21]. This finding illustrates that the TKFC–MDA5 interaction is not static but can itself be dynamically modulated, in this case by a noncoding RNA acting in a disease-specific context.

These results also indicate that TKFC can be considered a moonlighting protein, belonging to a group of metabolic enzymes that have acquired secondary regulatory functions in stress-response and innate immune signaling pathways. In this context, the modular K/L organization of TKFC and the preservation of signaling activity within the catalytically inactive K domain suggest the evolutionary repurposing of preexisting structural elements for regulatory functions independent of catalysis. Nevertheless, although evidence from disease-relevant cellular systems has begun to support a direct immunomodulatory role, further work will be required to determine whether this function operates similarly across physiological, non-malignant settings.

## 8. Emerging Metabolic and Stress-Associated Functions of TKFC

In addition to its role in innate immunity, evidence links TKFC to a range of metabolic and stress-associated processes. The evidence supporting these associations is heterogeneous and uneven in strength, ranging from direct experimental demonstrations to purely correlative transcriptomic associations and disease-specific screens in which TKFC was not functionally characterized. Below, these findings are grouped by biological context and weighted according to the directness of the supporting evidence; whether they reflect a single underlying mechanism or several independent facets of TKFC biology is revisited in Section 11.

Loss of TKFC redirects carbon flux toward oxidative and stress-associated pathways, as detailed in Section 5 [27]. Thus, TKFC appears to function not merely as a fructolytic enzyme but also as a determinant of fructose-derived metabolic flux and cellular redox balance during fructose metabolism.

A second, related facet concerns the handling of reactive trioses. Beyond its established metabolic role, TKFC-catalyzed phosphorylation of DHA to DHAP (Section 5) may contribute to the clearance of chemically reactive triose intermediates capable of promoting glycation and oxidative stress. Support for such a protective function comes largely from non-mammalian systems. In *Saccharomyces cerevisiae*, DHA kinase activity protects against the accumulation of reactive trioses: loss of DAK activity increases sensitivity to DHA toxicity, whereas its overexpression enhances tolerance [36]. In trypanosomatids, *Trypanosoma cruzi* possesses an ATP-dependent DHA kinase related to mammalian TKFC and tolerates high DHA concentrations, whereas *T. brucei*, which lacks a recognizable DHA kinase, is markedly DHA-sensitive [89]. The relevance of exogenous DHA is not restricted to experimental systems, because human exposure also occurs through cutaneous contact with widely used sunless tanning products, with additional inhalation exposure possible during spray tanning. In mammalian cells, exogenous DHA induces oxidative stress, mitochondrial dysfunction and advanced glycation-associated responses resembling those of excessive fructose metabolism [90]. More recent work showed that low-millimolar DHA exposure also induces cell-type-dependent DNA adduct formation and mutagenesis, with chromosomal aberrations and altered ploidy in bronchial and hepatic cell models [91]. Although TKFC protein abundance varied among the lung, cardiac and liver cell models examined, it did not correlate clearly with DHA cytotoxicity, leaving unresolved whether TKFC-mediated phosphorylation modulates these genotoxic responses. Furthermore, phosphorylation does not necessarily abolish carbonyl stress: the products formed by TKFC from DHA and D-glyceraldehyde—DHAP and glyceraldehyde 3-phosphate, respectively—can generate methylglyoxal through spontaneous phosphate-elimination reactions or as a minor side reaction of triose-phosphate isomerase [92]. Under conditions of excessive fructolytic flux, an increased triose-phosphate burden may therefore favor methylglyoxal formation, providing a potential link between fructose metabolism and advanced glycation [93]. Thus, TKFC may limit the accumulation of free reactive trioses while channeling their carbon into a triose-phosphate pool that can itself become a source of carbonyl stress when downstream metabolism is overloaded. Whether TKFC activity ultimately limits or redistributes carbonyl stress in mammalian cells may therefore depend on metabolic context and flux.

DHAP has been identified as a glycolytic metabolite that signals glucose availability to mTORC1, linking triose-phosphate metabolism to nutrient sensing [28]. During normal fructolysis, DHAP is generated by aldolase rather than by TKFC, which instead phosphorylates glyceraldehyde; a direct TKFC contribution to this signaling axis would therefore require an alternative route for DHAP generation. Experimentally, exogenous DHA has been used as a membrane-permeable precursor of DHAP through TKFC-mediated phosphorylation. In glucose-starved cells expressing TKFC, this approach was sufficient to raise intracellular DHAP and activate mTORC1 [28]. By contrast, in glucose-replete HepG3 liver cells, prolonged exposure to a cytotoxic concentration of DHA disrupted mTOR/AKT signaling, reduced glycolysis and oxidative phosphorylation, and induced mitochondrial reactive oxygen species, illustrating that exogenous DHA can elicit metabolic stress rather than simple nutrient signaling under high-load conditions [94]. More recently, the same strategy was used to investigate DHAP-dependent activation of a sterile alpha and TIR motif-containing protein 1 (SARM1)–cyclic ADP-ribose (cADPR)–Ca^2+^ signaling cascade leading to mitochondrial reactive oxygen species production and cellular senescence [95]. These studies establish TKFC-mediated phosphorylation of exogenous DHA as a useful experimental approach for selectively increasing intracellular DHAP but do not demonstrate that this reaction contributes physiologically to DHAP-dependent signaling in mammalian cells, for which no major endogenous source of DHA has yet been established.

Several transcriptomic and functional-screening studies place TKFC-associated pathways within programs of adaptive cellular remodeling, though none establishes a direct mechanistic role. Mouse *Tkfc* was identified among repair-associated macrophage genes during skeletal muscle regeneration [96] and the human gene emerged in kinome-wide CRISPR screens of selective autophagy—more prominently in the preprint than in the final report, where it was not functionally characterized [97,98]. Together, these observations support an association between TKFC-related metabolism and stress-responsive cellular programs but do not establish a direct mechanistic role for TKFC.

At the organ level, transcriptomic studies also associate TKFC with metabolic stress programs. In kidney, *Khk* and *Tkfc* are preferentially expressed in proximal tubule cells of rat and human nephrons [99], and in diabetic Akita mice *Tkfc* is coordinately upregulated with *Khk* and *Aldob* within a program spanning monosaccharide and NAD(H) metabolism, autophagy and stress responses; the glucagon-like peptide-1 (GLP-1) receptor agonist semaglutide reduced expression of all three enzymes while partially normalizing these stress pathways [100]. In liver, *Tkfc* was nominated as a central node of a long non-coding RNA (lncRNA)-regulated network linked to triglyceride accumulation in insulin-resistant HepG2 cells [101] and, in toxicogenomic analyses, as one of a minimal gene set predictive of early liver necrosis [102]. More recently, exploratory proteomic analysis of mouse duodenum found a nominal decrease in TKFC abundance during 5-fluorouracil-induced intestinal mucositis and a partial recovery following administration of a fermentation-derived bioactive preparation. In that study, neither of these changes remained significant after correction for multiple testing [103]. Collectively, these tissue-specific observations remain associative and lack direct functional validation of TKFC.

Non-canonical regulatory functions are not unique to TKFC. Other enzymes of the Hers pathway have also been shown to perform functions extending beyond intermediary metabolism. KHK-A, an alternatively spliced isoform of KHK, has been reported to function as a protein kinase in addition to its role in fructose metabolism. More recently, KHK was shown to promote adaptive ER proteostasis during nutrient stress by supporting IRE1α–XBP1 signaling while limiting PERK-mediated apoptosis, independently of its canonical role in fructose catabolism [104]. Although no comparable mechanism has yet been demonstrated for TKFC, these findings reinforce the emerging concept that enzymes of the Hers pathway may perform regulatory functions extending beyond intermediary metabolism.

Recent studies of cancer-associated metabolic remodeling suggest that TKFC may influence ferroptotic susceptibility. Ferroptosis, an iron-dependent, lipid peroxidation-driven form of regulated cell death, is a well-established vulnerability in hepatocellular carcinoma (HCC), partly reflecting the liver’s central role in iron and lipid metabolism [22,23,24,25]. A computational model combining multiple layers of biological data has independently nominated *TKFC* as a candidate gene associated with HCC [105]. Consistent with broader suppression of hepatic fructose metabolism during malignant transformation, transcriptomic analysis of The Cancer Genome Atlas Liver Hepatocellular Carcinoma (TCGA-LIHC) cohort identified *TKFC* as consistently downregulated in HCC together with *SLC2A2*, *KHK* and *ALDOB* [106]. Moving beyond these transcriptomic associations, a recent study functionally identified TKFC as an endogenous regulator of ferroptosis in HCC [26]. Whether TKFC’s proferroptotic activity depends on its catalytic function remains an open question; nonetheless, these findings indicate that TKFC-dependent stress adaptation pathways, discussed throughout this section, may also extend to ferroptotic regulation in cancer contexts.

Other recent studies have also identified TKFC as a molecular target of natural compounds in metabolic disease and cancer. Curcumin was shown to interact directly with TKFC through complementary chemical-proteomic and biophysical approaches, and genetic manipulation of TKFC modified its metabolic effects in cellular and mouse models of MASLD [71]. Icaritin was likewise reported to interact with TKFC, and TKFC overexpression synergistically potentiated icaritin-induced ferroptosis and antitumor efficacy in HCC models in vitro and in vivo [26]. However, neither study employed TKFC variants capable of distinguishing the contributions of triokinase, FMN cyclase and non-catalytic functions, and neither examined FAD, FMN or cFMN metabolism. Given the potential for these natural compounds to act through multiple cellular targets, evidence of TKFC engagement from the cellular thermal shift assay (CETSA) or related target-interaction assays does not, by itself, establish that the full pharmacological response is mediated specifically by TKFC. Even where genetic manipulation supports a contribution of TKFC, additional targets and pathways cannot be excluded. Thus, although these studies support the involvement of TKFC in the reported pharmacological responses, the specific TKFC function responsible for these effects remains unresolved.

Two lung adenocarcinoma studies further incorporated *TKFC* into estrogen-metabolism- and metabolism-based prognostic signatures associated with survival, one of them reporting computational correlations between *TKFC* expression and predicted sensitivity to Fulvestrant and SR16157; these associations were derived from transcriptomic modeling and were not experimentally validated [107,108]. A subsequent transcriptomic study of laryngeal squamous cell carcinoma identified *TKFC* as an overexpressed component of a three-gene immune- and metabolism-related prognostic signature, together with *ARG2* and *PLCG1*. Higher *TKFC* expression was independently associated with poorer overall survival, although the predictive performance of the composite signature was modest and the specific contribution of *TKFC* was not functionally investigated [109].

Altogether, the evidence supports a role for TKFC in coordinating metabolic flux with cellular responses to stress. The strength of this evidence is uneven—from direct experimental demonstration, as in ferroptosis regulation, to purely correlative transcriptomic associations—and whether these functions converge mechanistically through metabolic flux allocation, reactive triose handling, ferroptotic susceptibility or stress-responsive signaling remains the central open question.

## 9. Functional Diversification Through Alternative Splicing and Tissue-Specific Expression

In addition to its catalytic and regulatory versatility, *TKFC* undergoes transcript-level diversification through alternative splicing. The human *TKFC* gene (chromosome 11q12.2) comprises 18 exons, with an open reading frame extending from exon 2 to exon 18 that encodes the 575-residue bifunctional protein (isoform X6) endowed with both triokinase and FMN cyclase activities [2,11]. Beyond transcripts encoding this canonical enzyme, alternative processing of the last intron (intron 17) generates structurally distinct isoforms. Use of a weak alternative 3′-splice site retains a 56-nucleotide insertion between exons 17 and 18 (the *tkfc_ins56*-type splice), which introduces an in-frame stop codon and yields a shorter, 534-residue protein (isoform X8). This variant lacks the 50 C-terminal residues encoded by exon 18—including the L-domain elements required for FMN cyclase activity and the 535–554 mobile loop that carries the catalytically essential Arg543—and instead terminates in a short intron-encoded peptide. Recombinantly expressed isoform X8 is devoid of both triokinase and FMN cyclase activity (below 3% of the active isoform), confirming that this splicing event abolishes catalysis [110]. Because the premature stop lies within the last exon, the corresponding transcripts are predicted to escape nonsense-mediated decay, consistent with their detection in human tissues.

The two intron-17 splice types are differentially represented across human tissues. In multiple-tissue Northern blots probed with splice-specific oligonucleotides, the liver preferentially expresses the catalytically competent *tkfc*-type transcript, whereas the heart preferentially accumulates the alternative *tkfc_ins56*-type splice. Transcript-level quantification in Genotype-Tissue Expression (GTEx) independently supports the heart’s bias toward the *tkfc_ins56*/X8-type transcript (≈3.3-fold over the X6-type); more broadly, the X8-type transcript exceeds the X6-type by 1.6–3.9-fold across the tissues examined, although the Northern and RNA-seq datasets do not fully correlate [110]. Importantly, these splice-resolved patterns do not track global *TKFC* expression: in tissues such as intestine, liver and kidney, most (>90%) of the *TKFC* RNA-Seq signal derives from short, largely noncoding transcripts unrelated to the X6- or X8-coding mRNAs [110]. Overall *TKFC* transcript abundance therefore cannot be used as a proxy for the abundance of catalytically competent TKFC or for the balance between active and inactive protein isoforms. This is worth bearing in mind when performing transcriptome mining of *TKFC* gene.

Other observations hint that TKFC-associated pathways may also contribute to specialized cellular functions outside classical metabolic tissues. Proteomic analyses of human sperm identified a positive correlation between TKFC abundance and sperm motility, while a protein signature including TKFC and RUVBL1 displayed diagnostic potential for oligoasthenozoospermia [111]. Although the mechanistic basis of this association remains unknown, it leaves open whether TKFC contributes to specialized metabolic or regulatory functions in male germ cells.

The region removed in isoform X8 lies within the C-terminal L domain, leaving the N-terminal K domain intact. Since the isolated K domain is sufficient to inhibit MDA5-mediated antiviral signaling [15], preservation of this domain in the catalytically inactive X8 isoform raises the possibility that X8 could retain this immunoregulatory function. This inference is based on preservation of the N-terminal K domain and on experiments performed with the isolated K domain; no published cellular studies have demonstrated MDA5 suppression by the X8 isoform itself. Although this remains experimentally untested, it suggests that alternative splicing may separate immune regulation from catalysis, providing another mechanism for functional diversification. Alternative splicing is recognized as a mechanism for functional diversification, generating protein isoforms with distinct activities. Recent studies have shown that alternatively spliced variants of the metabolic enzyme PFKFB4 acquire non-canonical signaling functions through direct activation of AKT1 [112], whereas an alternatively spliced isoform of uromodulin relocalizes to mitochondria and promotes metabolic adaptation to cellular stress [113]. Similar principles may underlie the functional diversification of TKFC splice variants. TKFC therefore appears not simply as a metabolic enzyme but as a modular protein whose biological roles may be shaped by alternative splicing, tissue-specific splice-isoform expression and the partial separation of catalytic and regulatory functions.

## 10. Human Disease Associated with *TKFC* Variants

*TKFC* variants have been associated with a growing spectrum of human disorders including multisystem developmental disease, congenital cataracts, cardiomyopathy, immunological abnormalities, hereditary hypotrichosis and oculocutaneous albinism type 1 [29,30,31,32]. The clinical diversity of these phenotypes indicates that TKFC deficiency cannot be explained solely by impaired fructose metabolism.

The first genetically characterized patients with TKFC deficiency presented with congenital cataracts accompanied by variably severe multisystem manifestations including developmental delay, cardiomyopathy, hepatic dysfunction, anemia and neurological abnormalities. Some affected individuals also displayed elevated fibroblast growth factor 21 levels suggestive of mitochondrial stress responses [29]. Dietary fructose restriction did not consistently improve clinical manifestations, indicating that the disease phenotype extends beyond defective fructose utilization. Subsequent high-resolution inner-ear imaging of one of these patients revealed a symmetric cochlear apical malformation characterized by an additional half-turn and increased cochlear height. The patient presented with balance difficulties and sound hypersensitivity but no hearing loss, and the clinical significance of this isolated radiological observation remains uncertain [114].

Several TKFC-associated phenotypes overlap clinically with Sengers syndrome and related mitochondrial disorders characterized by congenital cataracts, skeletal and cardiac myopathy, lactic acidosis and defective energy metabolism [115]. Originally described on clinical and histopathological grounds decades before its molecular basis became accessible, Sengers syndrome subsequently proved to encompass considerable genetic and biochemical heterogeneity [115,116,117]. This heterogeneity now extends to clinically related disorders involving acylglycerol kinase (AGK), adenine nucleotide translocator 1 (ANT1/SLC25A4) and TKFC [29,30,31]. Earlier investigations of Sengers and Sengers-like syndromes had already revealed mitochondrial dysfunction—involving altered adenine nucleotide transport and defective oxidative phosphorylation despite partially preserved routine respiratory-chain measurements [116,117]—anticipating the recognition that TKFC-associated disease likely reflects broader disturbances of metabolic adaptation and mitochondrial homeostasis rather than isolated impairment of fructose catabolism.

Structural and mutational studies provide a mechanistic framework for understanding several disease-associated variants (see Section 4). Mapping the reported variants onto the TKFC sequence reveals that they are not scattered along the chain but form two clusters, one in each domain (Figure 6). Gly192Arg, Val200Ile and Arg228Trp lie in the N-terminal K domain, near the triose pocket and His221 [30,32], whereas Gly445Ser, Gly542Arg and Arg543Ile lie in the C-terminal L domain, near the ATP site and within or beside the mobile loop 535–554 [29,31]. All are missense changes converging on the composite active site from either domain. The functional consequences of the characterized variants are summarized in Table 2.

The coexistence of metabolic, mitochondrial, immunological and developmental phenotypes across these patients suggests heterogeneity beyond what a single enzymatic defect would predict. Distinct classes of TKFC-associated disorders may therefore reflect different combinations of catalytic impairment, altered conformational dynamics and disruption of protein–protein interactions. This dissociation is underscored by the biochemically characterized variants, which markedly impair triokinase activity yet map to distinct domains and produce different phenotypes: N-terminal Gly192Arg and Arg228Trp with isolated hypotrichosis [30], C-terminal Gly445Ser and Arg543Ile with multisystem disease [29], and C-terminal Gly542Arg with isolated immunodeficiency [31]. The last two are adjacent residues within the same mobile loop, yet one causes a severe multisystem disorder and the other an immunodeficiency confined to the immune system. Phenotype therefore correlates with neither the domain affected nor the identity of the catalytic activity lost, suggesting that additional determinants such as tissue-specific expression, variant-specific effects on protein–protein interactions, and genetic background may contribute. However, effects of these patient-derived variants on MDA5 binding or other protein–protein interactions have not been experimentally demonstrated.

In summary, the expanding disease spectrum associated with *TKFC* variants supports disease mechanisms beyond impaired fructose metabolism. The marked phenotypic heterogeneity observed across patients is more consistent with differential disruption of interconnected catalytic, structural and regulatory properties than with impairment of a single metabolic reaction. This interpretation is consistent with the diverse biological functions reviewed here, including stress adaptation, innate immunity and mitochondrial homeostasis.

The strength of the genetic evidence is not yet uniform across the reported disease spectrum. Whereas multisystem developmental disease is supported by multiple independent families together with functional characterization of the corresponding variants, other reported associations remain based on more limited clinical evidence and will require further genetic and functional validation.

## 11. Conclusions

The functional landscape of TKFC that emerges from this review is summarized in Figure 7. TKFC was initially characterized as a fructolytic enzyme involved in triose phosphorylation, but its later identification as the mammalian FMN cyclase revealed a much broader and mechanistically distinctive biological role. Structural and biochemical studies subsequently supported a model in which TKFC is a conformationally dynamic bifunctional homodimer in which two distinct catalytic activities—triokinase and FMN cyclase reactions—are coordinated through composite intersubunit active sites and partially overlapping structural determinants. In this respect, TKFC represents an example of catalytic multifunctionality emerging within a conserved metabolic scaffold.

Over recent years, the biological scope of TKFC has expanded beyond classical fructose metabolism. Increasing evidence links TKFC-dependent pathways to oxidative stress adaptation, nutrient sensing, innate immune regulation, ferroptosis-associated processes and mitochondrial homeostasis. The growing diversity of phenotypes associated with pathogenic *TKFC* variants, and the existence of catalytically inactive splice isoforms retaining structurally important domains, together imply that TKFC biology cannot be understood solely in terms of intermediary metabolism. Instead, TKFC increasingly appears less as an isolated metabolic catalyst and more as a multifunctional node integrating carbon metabolism, cellular homeostasis and regulatory signaling pathways.

## 12. Future Directions

Future studies of TKFC should progress across several complementary levels, from structural definition of its catalytic states to direct investigation of its functions in human disease. At the structural level, a major priority is to obtain high-resolution experimental structures of full-length human TKFC in multiple catalytic states. Structures representing open and closed conformations, with appropriate substrates, products and metal ions, would allow direct testing of the K/L-domain movements inferred from homology modeling and molecular dynamics simulations and would clarify how the same composite active-site architecture supports triokinase and FMN cyclase activities. The catalytic mechanism of FAD cyclization also requires further structural clarification, particularly regarding transition-state geometry and the individual contributions of active-site residues distinct from those governing triokinase activity. Such studies should also define the structural basis of the distinct Mg^2+^ versus Mn^2+^/Co^2+^ requirements and determine whether metal-dependent switching between catalytic modes can operate under physiologically relevant conditions.

At the biochemical and cellular level, separation-of-function approaches provide a particularly important opportunity to disentangle the multiple activities of TKFC. Variants that abolish or strongly impair triokinase activity while retaining FMN cyclase activity, together with substitutions that disrupt both reactions, should be combined with cellular complementation and targeted metabolomic analyses. In particular, sensitive measurement of FAD, FMN and cFMN will be required to determine whether TKFC contributes measurably to cFMN formation in living mammalian cells. Although cFMN occurs endogenously and purified enzymes capable of its synthesis and hydrolysis have been characterized, the existence of an integrated physiological cFMN turnover pathway remains unproven. Identifying the phosphodiesterase isoform or isoforms responsible for cFMN degradation in vivo, as well as cFMN-binding proteins and downstream effectors, will therefore be essential for establishing whether flavin cyclization has a specific physiological function or primarily reflects catalytic promiscuity of the TKFC scaffold.

A related priority is to distinguish the catalytic functions of TKFC from its non-catalytic regulatory activities. The effects of TKFC on MDA5-dependent signaling, stress adaptation and ferroptosis should be examined using separation-of-function variants rather than TKFC depletion or overexpression alone. This approach will be particularly important for determining whether these phenotypes depend on triokinase activity, FMN cyclase/cFMN production, or protein–protein interactions independent of either catalytic reaction. The same strategy should help define the functional significance of catalytically inactive splice isoforms and clarify whether preservation of the K domain is sufficient to maintain regulatory functions in their native cellular context.

At the organismal level, tissue-specific conditional knockout models and separation-of-function knock-in animals will be required to determine which TKFC activities are relevant in different physiological settings. Such models could distinguish the contributions of triokinase, FMN cyclase and non-catalytic functions in intestine, liver, kidney and other tissues while avoiding the interpretative limitations of constitutive loss of the entire protein. They would also provide an experimental framework for testing whether physiological or nutritional changes in manganese availability can modulate TKFC-dependent FMN cyclase activity in vivo.

An additional unresolved metabolic question concerns the origin and physiological relevance of free DHA in mammalian cells. Beyond its established role in fructose-derived glyceraldehyde metabolism, TKFC efficiently phosphorylates DHA, yet no major endogenous source of free DHA has been established. Low-level dephosphorylation of DHAP by promiscuous phosphatases represents one possible source, but its occurrence and quantitative importance in vivo remain unknown. Establishing the origin, concentration and metabolic fate of endogenous DHA will be necessary to determine whether DHA phosphorylation by TKFC contributes physiologically to reactive-triose clearance or other cellular processes beyond classical fructose metabolism.

Finally, studies in human biosamples should connect these mechanistic questions with TKFC-associated disease. Quantitative profiling of cFMN and related flavins in cells and tissues from individuals carrying pathogenic *TKFC* variants could establish whether altered flavin metabolism accompanies particular clinical phenotypes. Whenever possible, biochemical characterization of patient-derived variants should distinguish effects on triokinase activity, FMN cyclase activity, protein stability and protein–protein interactions rather than treating TKFC deficiency as a single functional state. Integrating these human studies with structural biology, targeted metabolomics and separation-of-function cellular and animal models should ultimately establish how the catalytic and regulatory properties of TKFC contribute to normal physiology and disease. More broadly, TKFC provides a useful model for understanding how metabolic proteins can evolve secondary regulatory functions and how catalytic, structural and signaling properties become integrated within a single multifunctional protein.

## Figures and Tables

**Figure 1:**
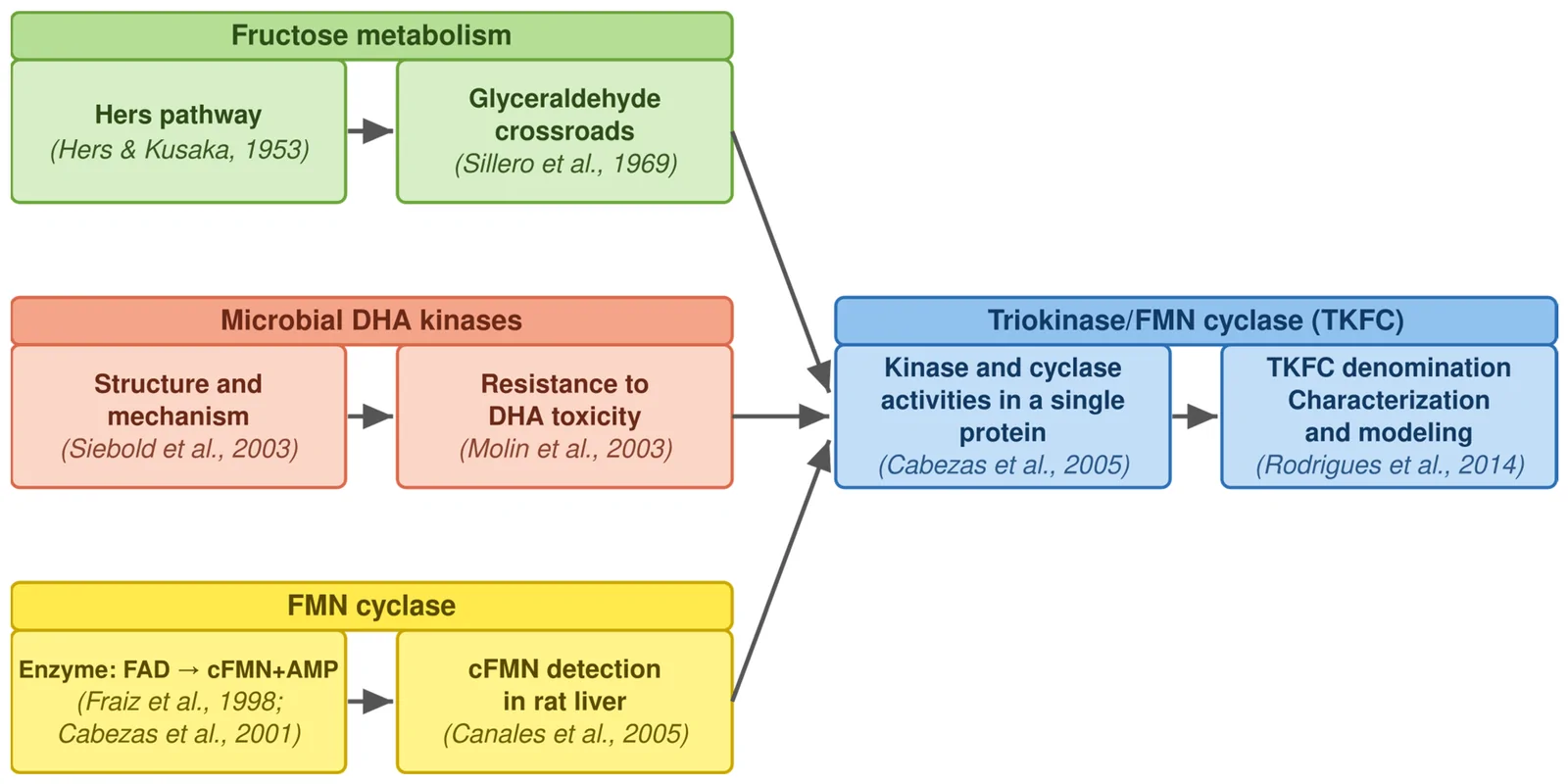
Historical convergence of research leading to the identification of TKFC. Three lines of research—hepatic fructose metabolism [3,5], microbial DHA kinase biology [7,8,36], and mammalian cyclic flavin metabolism [1,14,33]—converged with the demonstration that kinase and FMN cyclase activities reside in the same protein [2] and its subsequent characterization as TKFC [11].

**Figure 2:**
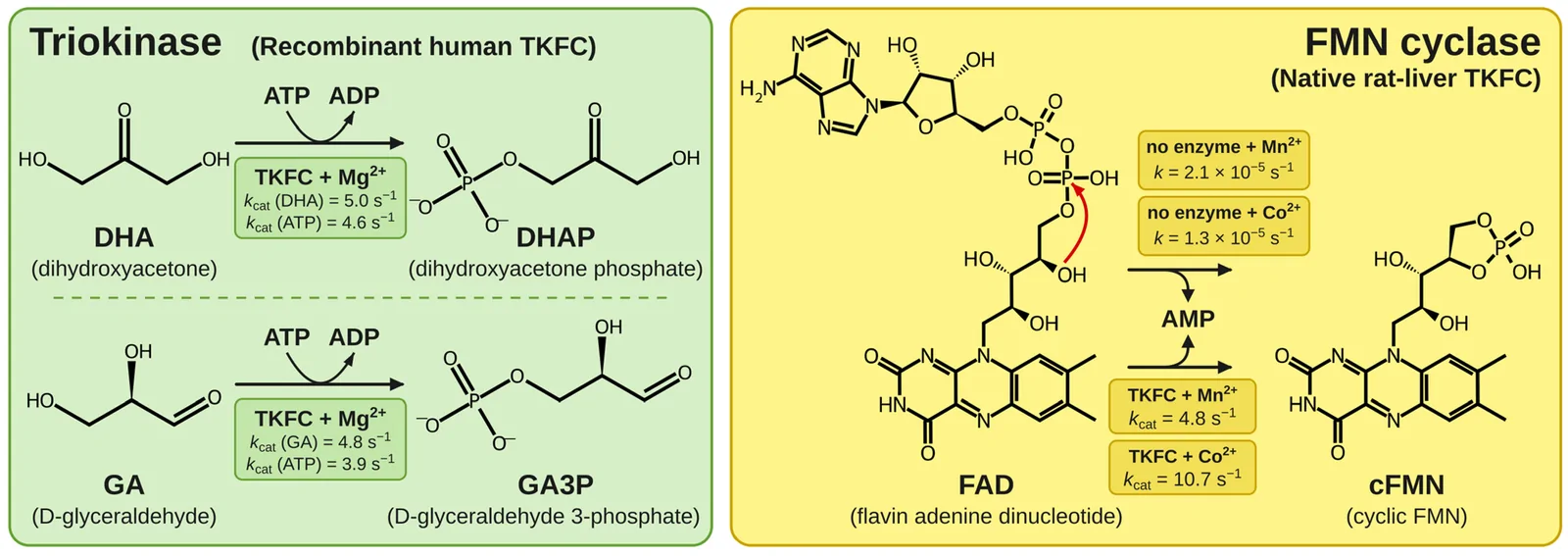
Triokinase and FMN cyclase reactions. (**Left**): ATP- and Mg^2+^-dependent phosphorylation of trioses by recombinant human TKFC. The *k*_cat_ values were determined at pH 7.5 and 37 °C [11]. (**Right**): Mn^2+^- or Co^2+^-dependent FAD cyclization in the absence or presence of native rat-liver TKFC. Labels show the non-enzymatic first-order rate constants (*k*) and enzymatic turnover numbers (*k*_cat_) also determined at pH 7.5 and 37 °C [14]. The values in the two panels derive from different enzyme preparations and are not intended for direct comparison. The red arrow indicates the intramolecular attack of the ribityl 4′-hydroxyl group on the proximal phosphate, yielding riboflavin 4′,5′-cyclic phosphate (cFMN).

**Figure 3:**
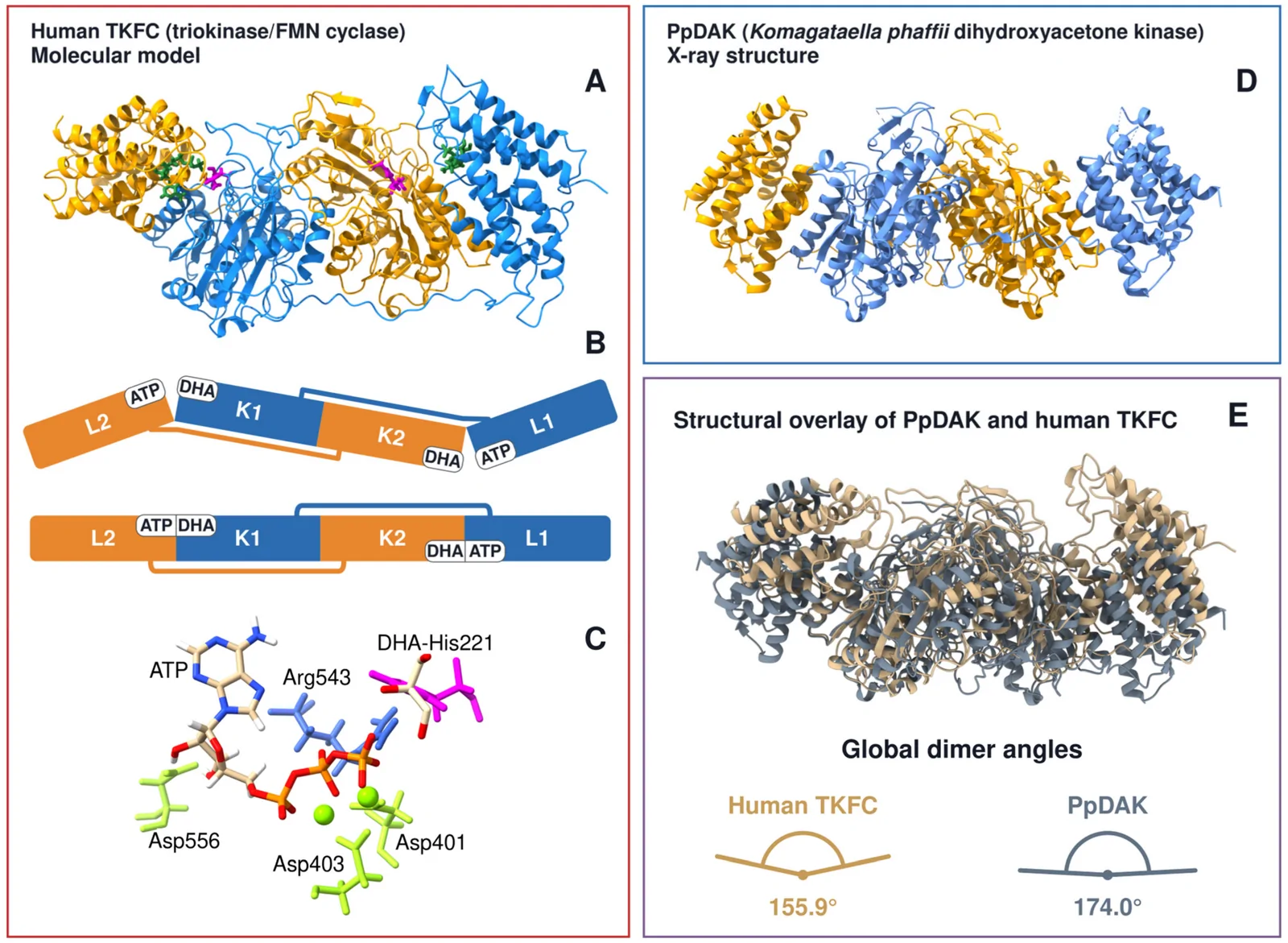
Homodimeric organization, catalytic architecture, and structural comparison of human TKFC and PpDAK. (**A**) Structural model of homodimeric human TKFC based on homology modeling and molecular dynamics simulations [30]. The two subunits are depicted in blue and orange, and the homodimer shows the two composite active sites (K1·L2 and K2·L1), with ATP in green and the DHA–His221 adduct in magenta. (**B**) Schematic of the conformational closure that brings ATP and DHA into proximity for phosphoryl transfer; this closure is required for triokinase but not for FMN cyclase activity. (**C**) Near-closed active site with DHA approaching a near-attack conformation; spheres represent Mg^2+^ ions. (**D**) PpDAK (*Komagataella phaffii* DHA kinase) experimental crystal structure (PDB 26VK) [46]. (**E**) Structural alignment of the complete PpDAK and human TKFC dimers based on a conserved core of 75 residue pairs (RMSD 0.985 Å), leaving peripheral divergence visible. Global dimer angles, defined from the centroids of the two peripheral domains and the central dimer core, are shown. The structural alignment, angle measurement and the molecular representations were prepared with UCSF ChimeraX 1.11.1 (https://www.rbvi.ucsf.edu/chimerax; accessed on 2 October 2026).

**Figure 4:**
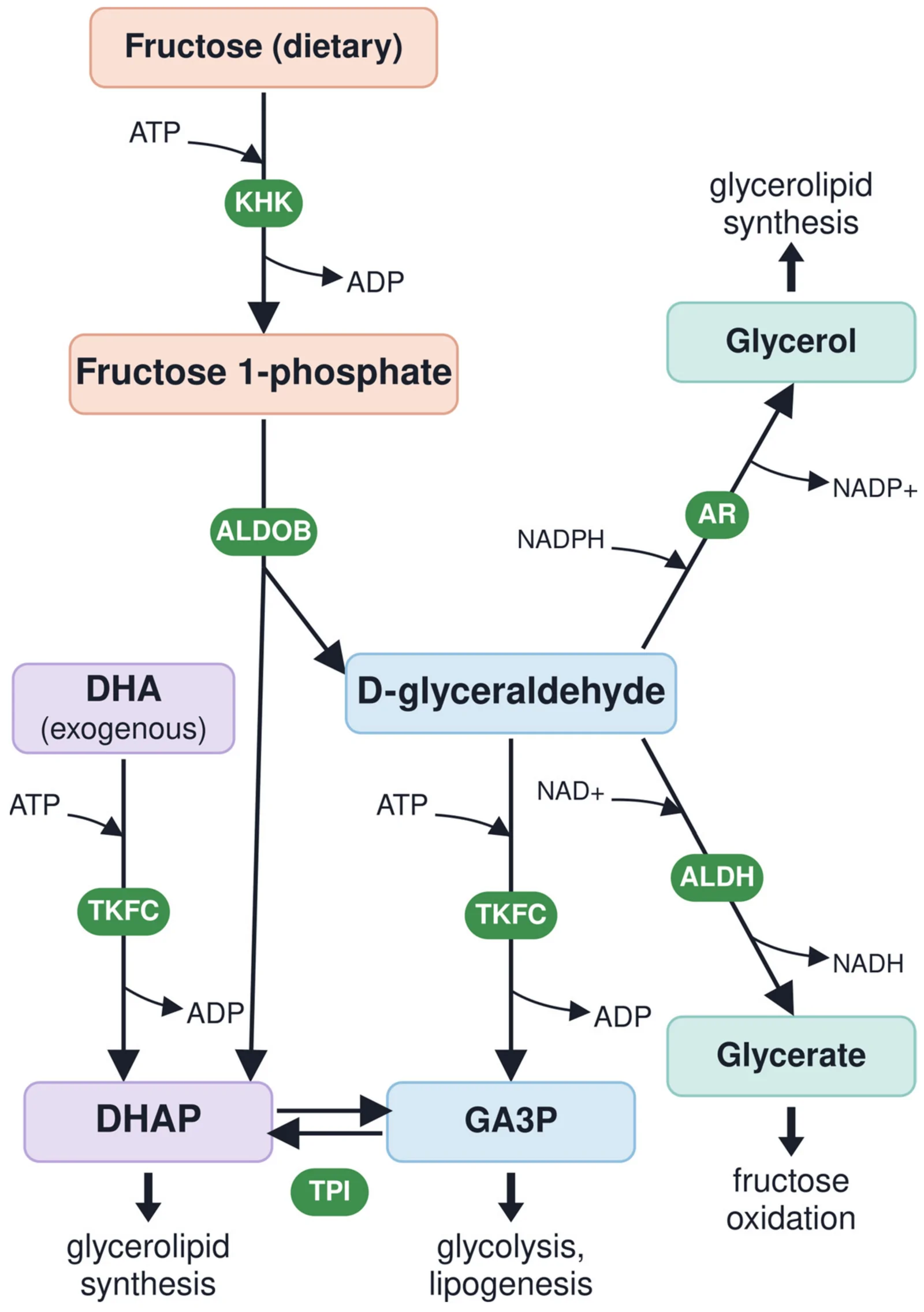
The Hers pathway and the glyceraldehyde metabolic crossroads. Fructose-derived D-glyceraldehyde can be phosphorylated by triokinase/FMN cyclase (TKFC) to D-glyceraldehyde 3-phosphate (GA3P) or enter alternative metabolic routes. TKFC also phosphorylates exogenous dihydroxyacetone (DHA) to dihydroxyacetone phosphate (DHAP). ALDH, aldehyde dehydrogenase; ALDOB, aldolase B; AR, aldose reductase; KHK, ketohexokinase; TPI, triose phosphate isomerase.

**Figure 5:**
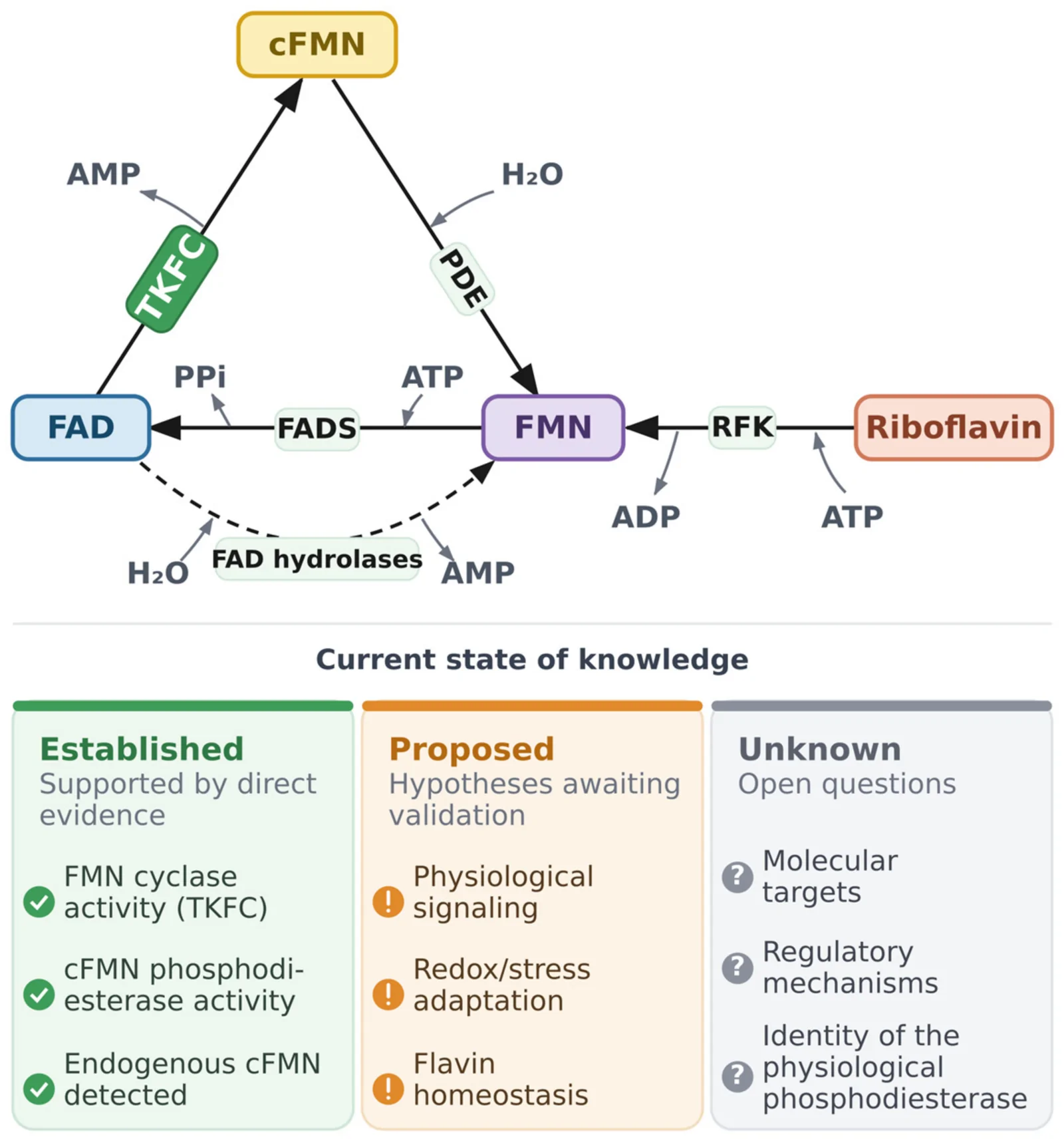
Current model of mammalian cFMN metabolism. Riboflavin, FMN, FAD and cFMN are linked by riboflavin kinase (RFK), FAD synthase (FADS), triokinase/FMN cyclase (TKFC) and cFMN phosphodiesterase (PDE), with a proposed FAD-hydrolase bypass shown by the dashed arrow. Individual enzymatic activities are established, but their integration into a physiological cFMN turnover cycle in vivo remains unproven.

**Figure 6:**
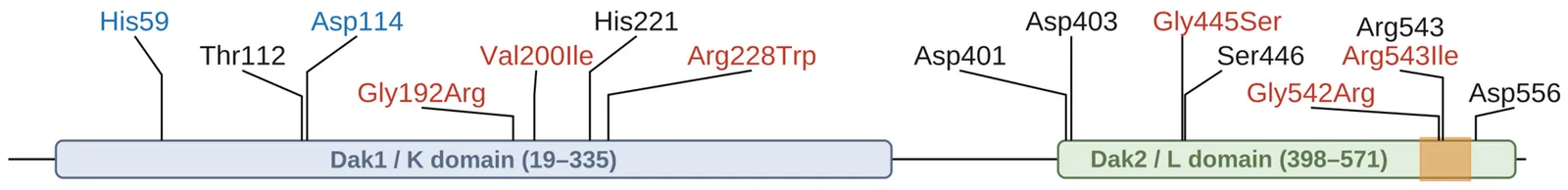
Residue map of the human TKFC subunit. Boxes indicate the Pfam domains Dak1/K (PF02733) and Dak2/L (PF02734); the orange band spanning residues 535–554 marks the mobile loop involved in active-site closure. Label colors denote residue class: red, disease-associated missense variants; black, residues characterized by site-directed mutagenesis; blue, proposed catalytic residues not yet experimentally tested. Associated phenotypes and the functional significance of the residues and variants are summarized in Table 2.

**Figure 7:**
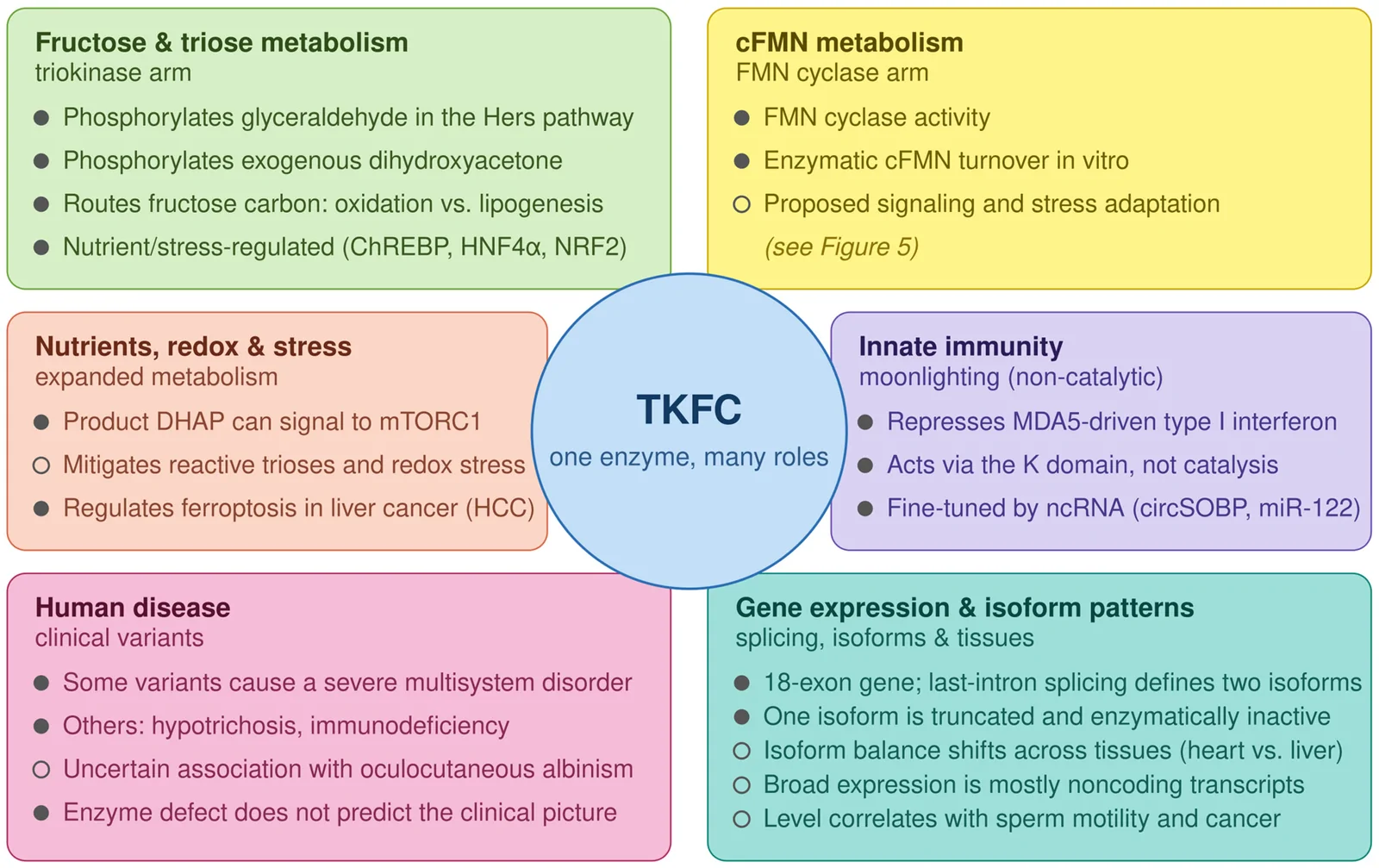
Overview of TKFC biology. The panels summarize the principal biological contexts discussed throughout this review. Within each panel, (●) denotes features supported by direct experimental evidence, whereas (○) indicates associations based primarily on transcriptomic, genetic or correlative studies.

**Table 1:** Steady-state kinetic parameters of mammalian TKFC.

Source *	Activity	Substrate	Metal Ion	*k*_cat_ (s^−1^)	*K*_m_ (µM)	*k*_cat_/*K*_m_ (M^−1^ s^−1^)
Human	DHA kinase	DHA	Mg^2+^	5.0	1.6	3.2 × 10^6^
Human	DHA kinase	ATP	Mg^2+^	4.6	43.2	1.1 × 10^5^
Human	GA kinase	GA	Mg^2+^	4.8	18.1	2.8 × 10^5^
Human	GA kinase	ATP	Mg^2+^	3.9	62.4	6.3 × 10^4^
Human	FMN cyclase	FAD	Mn^2+^	0.8	7.0	1.2 × 10^5^
Rat	FMN cyclase	FAD	Mn^2+^	4.8	8.8	5.5 × 10^5^
Rat	FMN cyclase	FAD	Co^2+^	10.7	114.0	9.3 × 10^4^

* Human values are from [11]; rat values are from [14].

**Table 2:** Functional annotation of selected TKFC residues.

Residue	Evidence Type	Triokinase	FMN Cyclase	Functionality Affected	Associated Phenotype	Reference
His59	Structural prediction	ND	ND	Catalysis (putative)	ND	[11]
Thr112	Site-directed mutagenesis	Reduced	Largely retained	Catalysis	ND	[11]
Asp114	Structural prediction	ND	ND	Catalysis (putative)	ND	[11]
Gly192Arg	Human patient variant	Abolished	Reduced	Triose binding/orientation	Hypotrichosis	[30]
Val200Ile	Human patient variant	ND	ND	Active-site closure (putative)	OCA1-associated syndrome *	[32]
His221	Site-directed mutagenesis	Abolished	Largely retained	Triose binding (covalent)	ND	[11]
Arg228Trp	Human patient variant	Abolished	Reduced	Triose binding	Hypotrichosis	[30]
Asp401	Site-directed mutagenesis	Abolished	Abolished	Metal coordination and nucleotide recognition	ND	[11]
Asp403	Site-directed mutagenesis	Abolished	Abolished	Metal coordination and nucleotide recognition	ND	[11]
Gly445Ser	Human patient variant	Reduced	ND	ATP/FAD recognition (putative)	Multisystem disease	[29]
Ser446	Site-directed mutagenesis	Reduced	Slightly reduced	Catalysis	ND	[11]
Gly542Arg	Human patient variant	Abolished	Largely retained	ATP binding and active-site closure	Immunodeficiency	[31]
Arg543	Site-directed mutagenesis	Abolished	ND	ATP positioning and phosphoryl transfer	ND	[11]
Arg543Ile	Human patient variant	Reduced	ND	Active-site closure (putative)	Multisystem disease	[29]
Asp556	Site-directed mutagenesis	Abolished	Abolished	ATP/FAD recognition	ND	[11]

ND, not determined. * OCA1, oculocutaneous albinism type 1. Reported in patients carrying biallelic variants in both *TYR* and *TKFC*; the specific contribution of TKFC remains uncertain.

## Data Availability

No new data were created or analyzed in this study. Data sharing is not applicable to this article.

## Abbreviations

The following abbreviations are used in this manuscript:

cFMN	riboflavin 4′,5′-cyclic phosphate
ChREBP	carbohydrate-responsive element-binding protein
CRISPR	clustered regularly interspaced short palindromic repeats
DAK	dihydroxyacetone kinase
DHA	dihydroxyacetone
DHAP	dihydroxyacetone phosphate
GA	D-glyceraldehyde
GA3P	D-glyceraldehyde 3-phosphate
HCC	hepatocellular carcinoma
MASLD	metabolic dysfunction-associated steatotic liver disease
MDA5	melanoma differentiation-associated protein 5
mTORC1	mechanistic target of rapamycin complex 1
PDB	Protein Data Bank
PTS	phosphotransferase system
RIG-I	retinoic acid-inducible gene I protein
RMSD	root-mean-square deviation
TKFC	triokinase/FMN cyclase
